# A low-cost, easy-to-use prototype bioreactor model for the investigation of human gut microbiota: validation using a prebiotic treatment

**DOI:** 10.3389/fmicb.2024.1250366

**Published:** 2024-05-08

**Authors:** Anne-Laure Agrinier, Geneviève Pilon, André Marette

**Affiliations:** ^1^Department of Medicine, Faculty of Medicine, Québec Heart and Lung Institute (IUCPQ), Université Laval, Québec, QC, Canada; ^2^Center for Nutrition, Health and Society (NUTRISS), Institute of Nutrition and Functional Foods (INAF), Université Laval Québec, Québec, QC, Canada

**Keywords:** gut model, bioreactor, gut microbiota, resistant starch, fecal fermentation

## Abstract

*In vitro* gut models allow for the study of the impact of molecules on human gut microbiota composition and function without the implication of the host. However, current models, such as the Simulator of Human Intestinal Microbial Ecosystem (SHIME^®^), are expensive, time-consuming, and require specialized personnel. Homemade *in vitro* models that lessen these issues have limited evidence of their humanlike functionality. In this study, we present the development of a low-cost and easy-to-use bioreactor with the proven functionality of human microbiota. In our model, we evaluated the capability of replicating human gut microbiota growth and the response of the human bacterial community to a prebiotic, resistant starch, particularly resistant starch type 2 (RS2). Our bioreactor produced an environment that was stable for pH, temperature, and anaerobic conditions. The bioreactor consistently cultivated bacterial communities over a 48 h time period, replicating the composition of the gut microbiota and the associated metabolite production response to RS2, in line with prior human studies. In response to the RS2 prebiotic, we observed an increase in *Bifidobacterium adolescentis* and *Bifidobacterium faecale* and an increase in the production of the short-chain fatty acids such as acetate, propionate, and isobutyrate. Taken together, these data demonstrate that our low-cost and user-friendly prototype bioreactor model provides a favorable environment for the growth of human gut microbiota and can mimic its response to a prebiotic.

## 1 Introduction

The importance of the intestinal microbiota for the overall health of the host has been highlighted over the past decades. Indeed, a growing body of literature indicates its involvement in the incidence of various disorders, such as type 2 diabetes and liver and inflammatory bowel diseases (Koh and Bäckhed, 2020; Hou et al., 2022). However, the composition of the gut microbiota is highly variable at the individual level (Donaldson et al., 2015). The bacterial composition of the gut microbiota is influenced by various factors such as diet, sex, age, and geographic location (Rothschild et al., 2018). Given the high inter-individual variability of the gut microbiota, responses to these modulating factors can differ substantially among individuals (Schloissnig et al., 2013). Dietary factors are known to strongly modulate the gut microbiota (Sonnenburg and Bäckhed, 2016). For example, in healthy individuals, consumption of dietary fibers such as whole-grain barley amplified short-chain fatty acid (SCFA)-producing strains and resulted in an increased ratio of *Prevotella*/*Bacteroides* (Myhrstad et al., 2020). Another dietary fiber, resistant starch, can reach the intestine without being digested by alpha-amylase and can be fermented by the gut microbiota. Resistant starch is considered a prebiotic because it has the potential to enhance the growth of beneficial bacteria and increase the production of metabolites, such as SCFAs, leading to beneficial effects on the health of the host (Scott et al., 2013; Gibson et al., 2017). There are several types of resistant starch, and, in particular, resistant starch type 2 (RS2) is defined as the native granular starch composed of ungelatinized granules (Maier et al., 2017). RS2 has been reported to change the composition of intestinal microbiota and SCFA production by promoting the growth of *Bifidobacterium adolescentis*, *Ruminococcus bromii*, and *Eubacterium rectale*, associated with an increase in acetate, butyrate, and propionate (Dobranowski and Stintzi, 2021). However, the response of the microbiota to resistant starch depends on the source of resistant starch, the age, and the baseline microbiota profile of the host (Bendiks et al., 2020). As the importance of the intestinal microbiota on health is increasingly recognized, more and more clinical or animal studies seek to unravel the role of the intestinal microbiota in its observed effects. There is therefore a growing need to study the role of the intestinal microbiota in the face of a treatment using a model that eliminates confounding host effects.

*In vitro* models are advantageous approaches in studying specific components of the diet. However, current models such as SHIME^®^ may not be accessible due to the high demands of time and energy to perform an experiment. Instead, batch *in vitro* models (bioreactors) can be an alternative approach. Batch *in vitro* models consist of a closed environment with culture media and microbiological biomass fermenting for 24–48 h without the addition of new culture media. These systems present many advantages, as they are reproducible, have no ethical limitations, have a controlled environment, and eliminate host influence (Brodkorb et al., 2019). However, limitations of these systems include the accumulation of microbial products and the fermentation time being restricted to 48 h, leading to the inability to maintain a stable microbiota community for more than 48 h. Furthermore, batch models are often employed for specific applications without pH control, and there is limited evidence supporting their functionality (Auchtung et al., 2015; Cherta-Murillo et al., 2023; Rachmühl et al., 2023). This study aims to develop a low-cost *in vitro* model that is user-friendly while monitoring important culture media parameters and ensuring a swift turnaround between experiments. The bioreactor is designed to be adaptable to various culture conditions, allowing the choice of pH, temperature, or oxygen concentration, thereby offering the possibility to emulate various parts of the gastrointestinal tract or different microbiota (specific bacteria, humans, or mice). Importantly, this model has demonstrated functionality with the human microbiota in response to a conventional prebiotic.

## 2 Materials and methods

### 2.1 Design of the colon model

**Figure 1:**
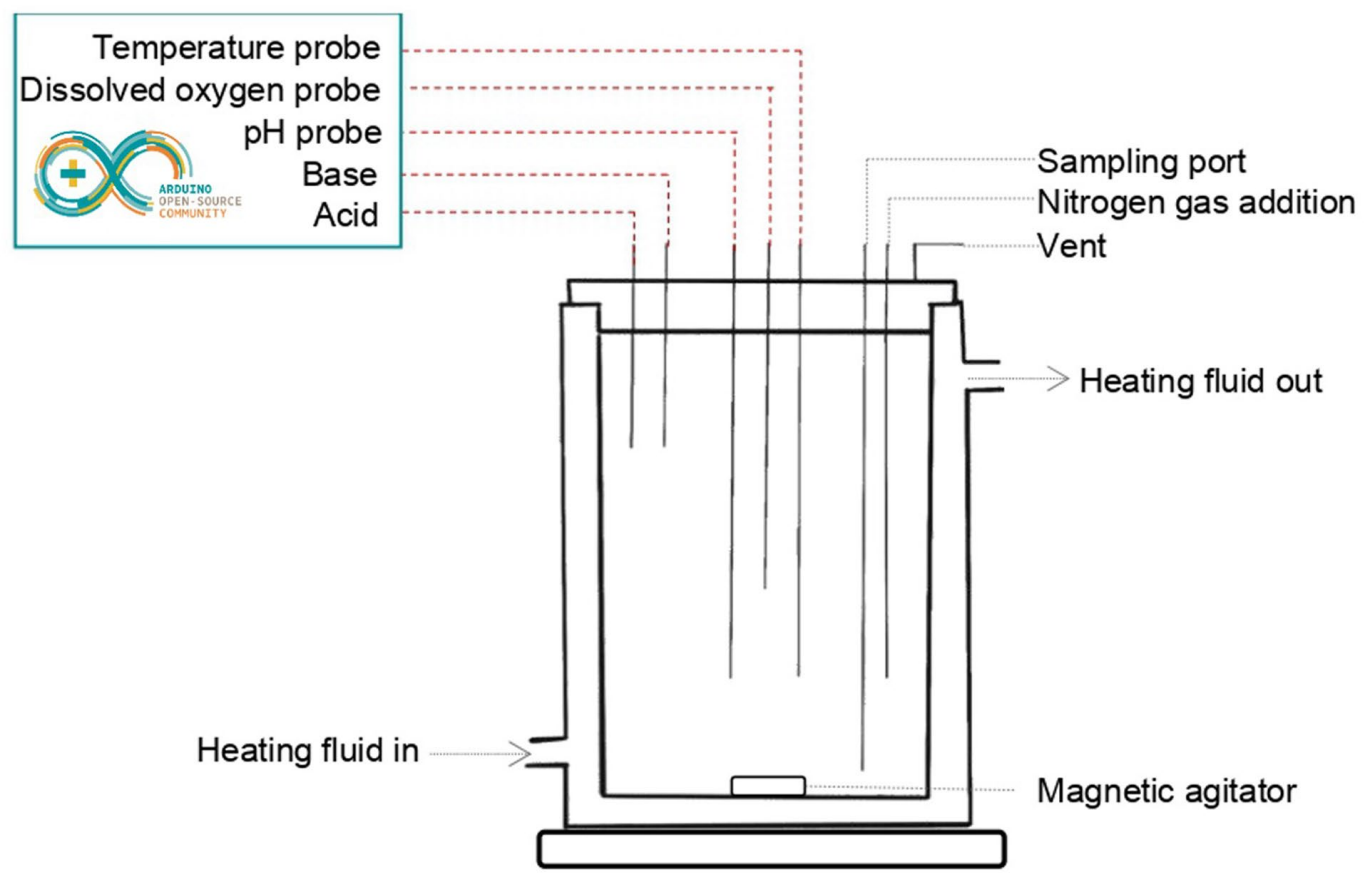
Schematic representation of the bioreactor module.

The colon model consisted of a 400-mL jacketed beaker and a control box, and the assembly is referred to as a bioreactor (Figure 1). The beaker cap was designed to have four input ports, one output port, and three ports for probes (pH, temperature, and dissolved oxygen), and was 3D printed in carbon-fiber nylon. Input ports were used for adding acid and base, taking samples, adding inoculum, and purging nitrogen gas to maintain anaerobic conditions in the beaker. The output port was used for gas venting to relieve pressure. Continuous agitation was maintained using a magnetic plate stirrer. The temperature was maintained at 37°C using a mix of ethylene-glycol:water (1,2, v,v) through the double wall of the beaker. The control box was used to monitor and adjust the temperature, the concentration of dissolved oxygen, and the pH using an Arduino Uno microprocessor and the ecosystem EZO^™^ (Atlas Scientific, NY, United States). The pH of the culture medium was maintained at a stable value of ±0.1 by the addition of acid or base using a system of two peristaltic pumps (Atlas Scientific, NY, United States), connected to a bottle of acid solution (HCl 0.1 N) and a bottle of a basic solution (NaOH 0.25 N), respectively.

### 2.2 *Bifidobacterium longum* preparation

To obtain working cultures of *Bifidobacterium longum* subspecies *longum* (ATCC 15707), pre-cultures were carried out. Frozen *B. longum* was added to 10 mL of brain heart infusion (BHI) media (*BD* Biosciences Franklin Lakes, NJ, United States) and was cultured under anaerobic conditions at 37°C for 24 h (pre-culture n°1). A second pre-culture (pre-culture n°2) was then performed by adding 1 mL of pre-culture n°1 to 9 mL of BHI media and growing it under anaerobic conditions at 37°C for 24 h. The pre-culture n°2 was used for fermentation in our model.

### 2.3 Fecal human inoculum preparation

To prepare the inoculum, we used a fecal sample from a single healthy individual. This individual was a 26-year-old woman with a BMI of 19 kg/m^2^ and had no history of GI disorders or antibiotic use for at least 6 months prior to the sample collection. Within 2 h of fecal sample collection, the sample was diluted in 0.9% saline solution (1:10, w:v) and glycerol (1,10, v:v) and was then filtered at 330 μm (Whirl-Pak^™^ Sterile Filter Bags, Fisher Scientific, Ottawa).

### 2.4 Tested product

Potato starch was purchased from Sigma-Aldrich Chemical Company (catalog no. S2004; St. Louis, MO), and 9 g of it were added to each bioreactor.

### 2.5 Culture media

Culture media for *B. longum* was prepared by dissolving 37 g of BHI powder (BD Diagnostics) in 1 L of distilled water, which was then autoclaved at 121°C for 1 h.

Culture media for human microbiota was based on Li et al. (2019). Briefly, the media was composed of 2.0 g L^−1^ of peptone water, 2.0 g L^−1^ of yeast extract, 0.5 g L^−1^ of L-cysteine hydrochloride, 2 mL L^−1^ of Tween 80, 5 mg L^−1^ of hemin, 10 μL L^−1^ of vitamin K1, 1.0 g L^−1^ of NaCl, 0.4 g L^−1^ of K_2_HPO_4_, 0.4 g L^−1^ of KH_2_PO_4_, 0.1 g L^−1^ of MgSO_4_7H_2_O, 0.1 g L^−1^ of CaCl_2_2H_2_O, 4.0 g L^−1^ of NaHCO_3_, 4.0 g L^−1^ of porcine gastric mucin, 0.25 g L^−1^ of sodium cholate, and 0.25 g L^−1^ of sodium chenodeoxycholate.

### 2.6 Culture conditions

For *B. longum* cultures, 10 mL of pre-culture n°2 were added to the culture media, maintaining a pH of 7.0 ± 0.1 and an oxygen concentration below 1 mg/L. The cultures of *B. longum* were grown in triplicate in identical bioreactors in parallel.

To reproduce human colon conditions, the fermentation parameters were fixed at a temperature of 37°C, a pH of 6.8 ± 0.1, and an oxygen concentration below 1 mg/L. After the stabilization of the media, 20 mL of fecal inoculum were added to achieve a 10% (v:v) ratio in the culture media. Both the control and RS2 conditions were executed in two identical bioreactors operated simultaneously. Each condition was replicated four times. To mitigate any potential bias stemming from the bioreactors, they were alternated between conditions.

### 2.7 Sampling

Sampling for *B. longum* cultures was performed at 0, 2, 6, 10, 22, and 24 h. For human microbiota cultures, samples were collected in the inoculum at 0, 24, and 48 h and were immediately centrifuged at 14,000 g for 8 min at 4°C. Supernatants were separated from pellets and were stored at −80°C for SCFA analysis while the pellets were stored at −80°C until DNA extraction.

### 2.8 Determination of bacterial viability

A measure of 50 μL of culture was diluted in 450 μL of PBS, and 1.25 μL of 20 mM propidium monoazide (PMA) solution (Biotium, Hayward, CA, United States) were added to the sample. The samples were shielded from light, agitated for 5 min, and then exposed to the **PMA-Lite**^**™**^ device (Biotium, Hayward, CA, United States) for 15 min. Finally, the samples were centrifuged at 14,000 g for 8 min at 4°C, and the pellets were stored at −80°C until DNA extraction.

### 2.9 DNA extraction

The pellets from *B. longum* cultures were used for DNA extraction with the ZymoBIOMICS^™^ DNA Miniprep Kit (Zymo Research, cat #D4300), following the manufacturer’s instructions.

Genomic DNA from bacterial cultures of human microbiota was extracted from the culture pellets of each sample using a DNA extraction kit (Qiagen QIamp DNA Stool Mini Kit, Qiagen, Valencia, CA, United States) following the manufacturer’s instructions with modifications. The samples were homogenized with 1 mL of Inhibitex (Qiagen, Valencia, CA, United States). Part of the lysate was transferred to tubes containing 0.1-mm zirconium beads and 50 mg of lysozyme. The tubes were incubated at 37°C for 1 h. The samples were homogenized two times for 1 min using a VWR bead mill. The suspension was heated for 5 min at 95°C. After centrifugation, DNA extraction of the samples was carried out following the manufacturer’s protocol. DNA concentrations were evaluated spectrophotometrically using a NanoDrop ND-1000 (Thermo Scientific). The extracted DNA was stored at −20°C until used.

### 2.10 qPCR

Quantitative real-time PCR (qPCR) was carried out using 4 μL of extracted DNA at a concentration of 0.25 ng/μL, 5 μL of Advanced Master Mix (Wisent Bioproducts), and 0.5 μL of each primer (diluted at a concentration of 10 μM) in a total reaction volume of 10 μL. The qPCR program settings were as follows: 95°C for 2 min, followed by 40 cycles of 95°C for 5 s, 53°C for 30 s, and 72°C for 20 s, ending with a melting curve ranging from 65 ° C to 95°C on the CFX96 Touch Real-Time PCR Detection System (Bio-Rad Laboratories). Primer sequences targeting 16S rDNA are shown in Supplementary Table S1.

### 2.11 16S rRNA amplicon sequencing

Libraries were sequenced on the Illumina MiSeq PE300 platform (Illumina, San Diego, CA, United States) using a 2 ×300 bp paired-end run. The forward primer “CCTACGGGNGGCWGCAG” and the reverse primer “GACTACHVGGGTATCTAATCC” were used for amplification. This yielded an average number of read pairs per sample of approximately 39,766. Forward and reverse primers were removed from 16S rRNA gene amplicons using Cutadapt (v3.4) (Martin, 2011). Sequence reads were analyzed using the DADA2 package (v1.22.0) (Callahan et al., 2016). Forward and reverse reads were first trimmed at 280 bp and 240 bp, respectively, to remove low-quality regions. The sequences with an expected error threshold of >2 and > 4 for the forward and reverse reads, respectively, with ambiguous bases, and with a quality score of less than 3 or equal to 2 were discarded. Dereplication and denoising of the filtered sequences were carried out using DADA2 default parameters. Denoised forward and reverse reads were merged (all reads with any mismatches were removed) and searched for chimeras. Taxonomic assignment of amplicon sequence variants (ASVs) was performed using the RDP classifier algorithm (v2.2), trained against the Silva database (version 138) (McLaren, 2020). A phylogenetic tree was built in R using the ape package (v5.6.2) (Paradis and Schliep, 2019). Data visualization and analysis were performed in R using the phyloseq package (v1.38.0) (McMurdie and Holmes, 2013). To quantify bacterial alpha diversity, Shannon and Simpson’s reciprocal indexes were calculated. Principal coordinates analysis (PCoA) was performed on an unweighted UniFrac distance matrix to measure beta diversity. The statistical significance of differentially abundant bacteria between the two distinct biological conditions was measured with LEfSe (Segata et al., 2011). A *p*-value of <0.05 and a linear discriminant analysis (LDA) score of ≥2.5 will be considered statistically significant.

### 2.12 SCFAs

SCFAs were quantified using gas chromatography. The supernatant from the cultures was collected and kept frozen at −80°C until extraction. The samples were diluted with Milli-Q water to reach standard curve values (2–20 dilutions). The supernatant was spiked with 4-methylvaleric acid and was acidified with phosphoric acid at 10%. To extract SCFAs, the samples were mixed for 2 min with an equal volume of diethyl ether and were then centrifuged at 18,000 g for 10 min at 4°C. Organic phase analysis was performed on a GC-FID system (Shimadzu), consisting of a GC 2010 Plus gas chromatograph equipped with an AOC-20s auto-sampler, an AOC-20i auto-injector, and a flame ionization detector. The system was controlled by GC Solution software. SCFAs were separated on a Nukol capillary GC column (30 m x 0.25 mm id, 0.25 μM film thickness, Supelco Analytical). The column flow was constant at 1.3 mL/min of hydrogen. The injector temperature was set at 230°C and the detector temperature was set at 250°C. The oven temperature was initially programmed at 60°C and then increased to 200°C at 12°C/min, which was held for 2 min. SCFAs were quantified using a 5-point calibration curve prepared with a mix of acetic acid, propionic acid, butyric acid, isobutyric acid, valeric acid, isovaleric acid, and internal standard 4-methyl valeric acid. Phosphoric acid was purchased from VWR (Mississauga, ON). Diethyl ether (99.5%) and all the 99% grade standards (acetic acid, propionic acid, isobutyric acid, butyric acid, isovaleric acid, valeric acid, and internal standard 4-methyl valeric acid) were purchased from Sigma-Aldrich (St. Louis, MO).

### 2.13 Statistical analyses

**Figure 2:**
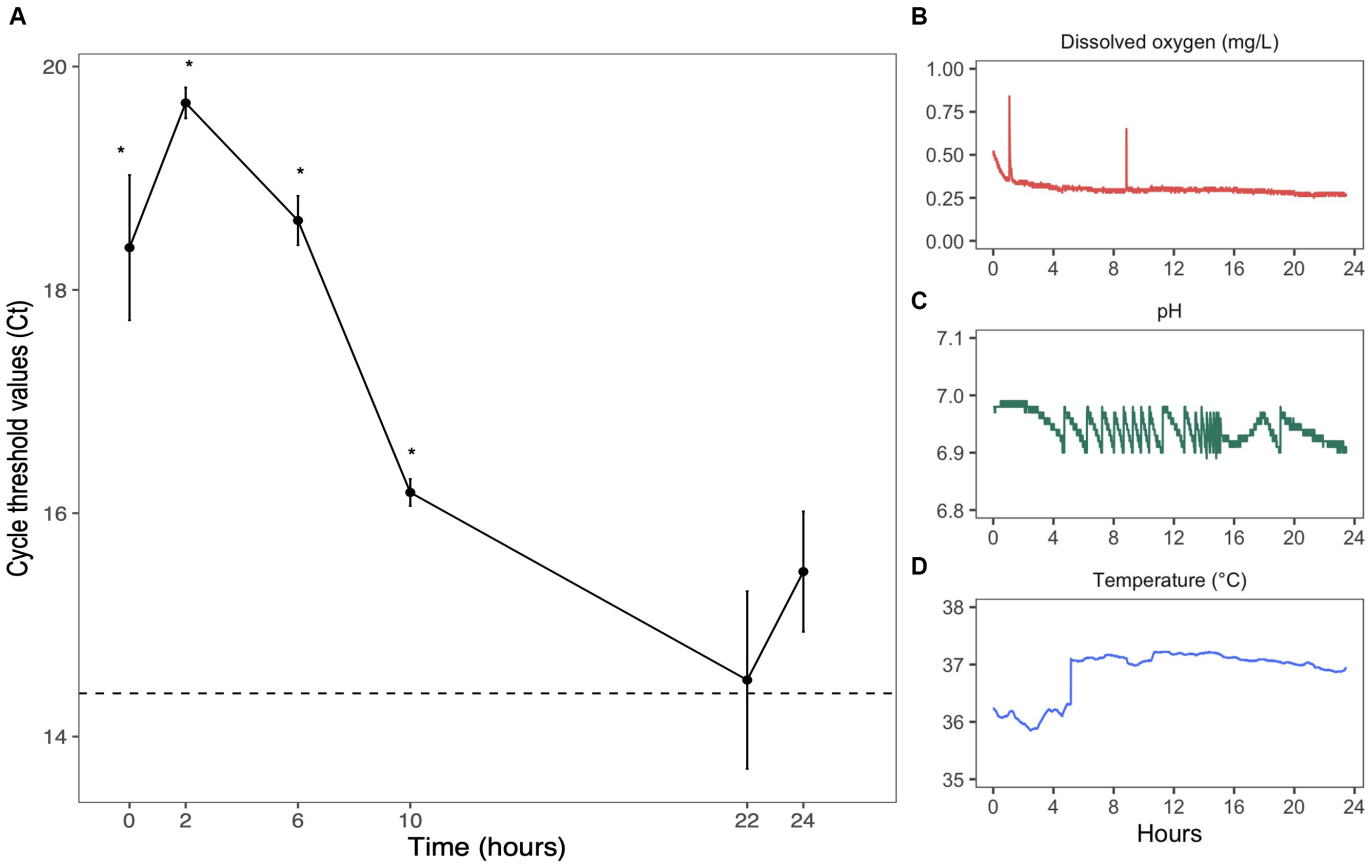
Anaerobic growth of *Bifidobacterium longum*. **(A)** *Bifidobacterium longum* quantity evaluated by qPCR. Each point represented the mean value of three replicates ± sem. Samples were analyzed in three technical triplicates. Dashed line represents Ct values found in *B. longum* starter. * = *p* < 0.05 in comparison to starter values. Evolution of parameters inside one culture over 24 h: **(B)** dissolved oxygen (mg/L), **(C)** pH, and **(D)** temperature (°C).

**Figure 3:**
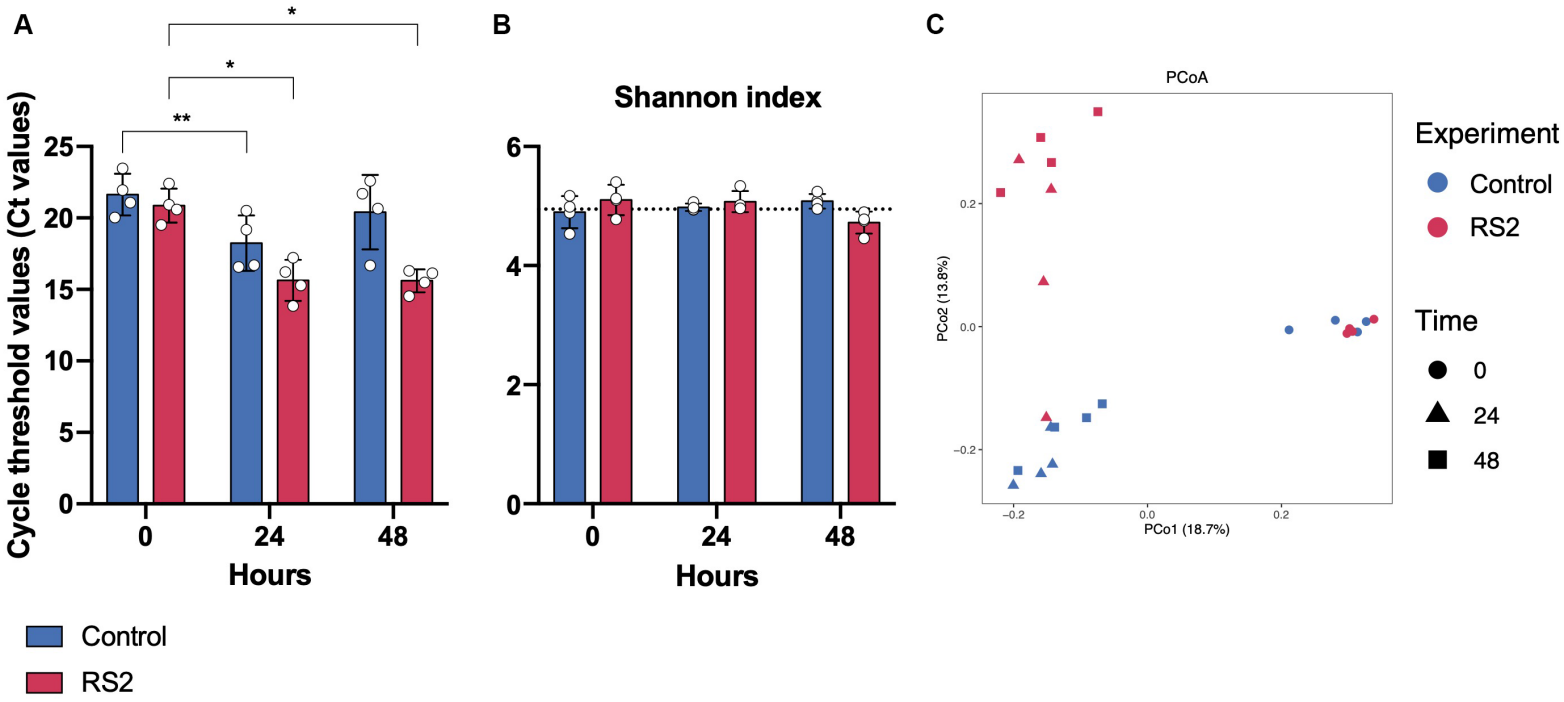
Impact of bioreactors and RS2 on growth and microbial diversity in our model. **(A)** Threshold values obtained by PMA-qPCR for viable bacteria. **(B)** Shannon alpha diversity index over 48 h of fermentation. Dot line represents the alpha diversity found in the inoculum. **(C)** Principal coordinate analysis (PCoA) using unweighted UniFrac distances of overall bacterial presence in bioreactor samples at 0, 24, and 48 h of fermentation. PERMANOVA test for control fermentations between time 0 vs. time 24 *p* = 0.023, time 0 vs. time 48 *p* = 0.032, time 24 vs. time 48 *p* = 0.235. PERMANOVA test between control vs. RS2 *p* = 0.599 at time 0, *p* = 0.024 at 24 h, and *p* = 0.03 at 48 h. **(A,B)** data are represented as means ± s.d. (*n* = 4). * = *p* < 0.05, ** = *p* < 0.01.

**Figure 4:**
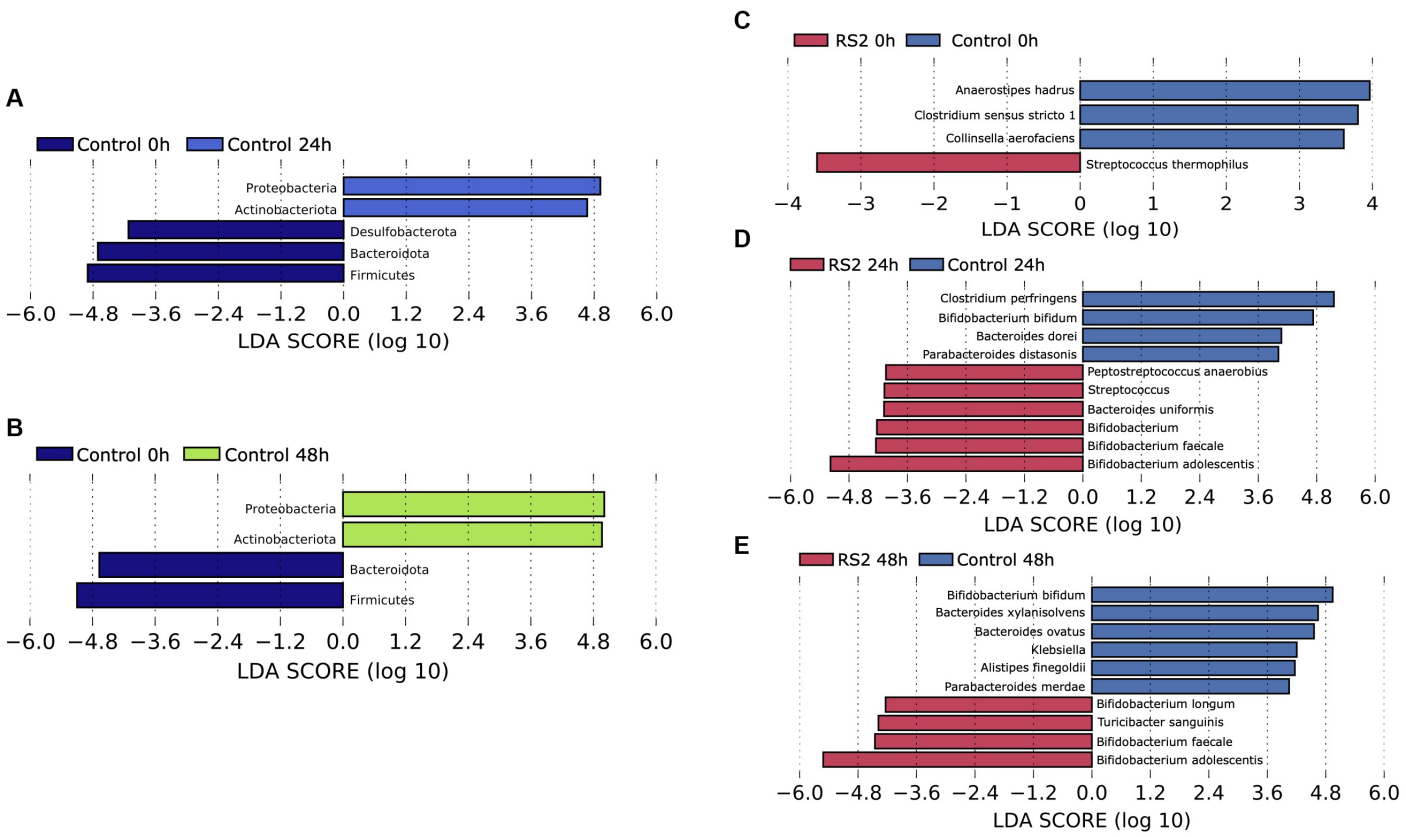
Evolution of the composition of bacterial communities. For control fermentations, LEfSe was used to assess the phylum that more strongly discriminates the composition of the microbiota after 24 h **(A)** and 48 h **(B)** of fermentation in comparison to the beginning. LEfSe was used to assess the genera that more strongly discriminate between the composition of the microbiota of the control and RS2 at time 0 **(C)**, time 24 **(D)**, and time 48 **(E)** of the study.

All statistical analyses were performed using R (v4.1.1; available from https://www.r-project.org/). Data are expressed as mean ± standard deviation (s.d.). A one-way ANOVA was used to assess the differences in means of the measured parameters over time (see Figures 2, 3). A two-way ANOVA followed by a Tukey HSD post-hoc test was used with treatment and time as independent factors to determine the effect of these factors and their interaction on growth, SCFA levels, and alpha diversity of the human microbiota (see Figures 3, 4). If the data did not follow a normal distribution, non-parametric tests, such as the Kruskal–Wallis test, were performed. A *p*-value of <0.05 was used to determine statistical significance.

## 3 Results

### 3.1 Validation of the anaerobic environment

An anaerobic environment is essential to replicate gut microbiota growth conditions. To assess the capacity of our model to provide an anaerobic environment, we monitored the growth of *B. longum* subspecies *longum*, a strict anaerobic bacterium, for 24 h in our bioreactor.

The growth of *B. longum* was verified by qPCR with specific primers (Figure 2A). The decrease in cycle threshold (Ct) values over 24 h suggested an increase in abundance, confirming the efficiency of our anaerobic system. After 22 h, Ct values reached levels found in the inoculum (the dashed line represents Ct values found in *B. longum* starter: pre-culture n°2) (Figure 2A). The controller adjusted the pH in response to culture growth and the release of organic acids, successfully maintaining it at approximately 7 through automated additions of base and acid (Figure 2C). Over 24 h of fermentation, the pH was kept at 6.94 ± 0.02 with a coefficient of variation of 0.3%, the dissolved oxygen level was maintained at 0.30 ± 0.03 mg/L (Figure 2B) with a coefficient of variation of 12.4%, and the temperature was kept at 36.9 ± 0.42°C (Figure 2D) with a coefficient of variation of 1.1%. Thus, pH, dissolved oxygen, and temperature remained stable over 24 h of culture, reflecting the environmental stability of our system.

### 3.2 Bioreactor culture capability with human gut microbiota

We then evaluated the ability of our bioreactor to grow intestinal human microbiota over 48 h in batch mode. We inoculated the bioreactor with human fecal material and started fermentation for 48 h under conditions (temperature, pH, and dissolved oxygen) resembling those found in the colon. The bioreactor allowed the growth of human microbiota for 24 h, as evidenced by the decrease in Ct values observed between 0 and 24 h. Subsequently, the growth was stabilized after 24 h of fermentation and was diminished at the 48-h mark, as depicted by the blue bars in Figure 3A. To identify the impact of our model on the bacterial community, we analyzed the composition of the gut microbiota by 16S rRNA high-throughput sequencing at the beginning of fermentation and after 24 and 48 h. As reflected by the Shannon index, the alpha diversity of the microbiota was not affected by the bioreactor (Figure 3B), highlighting the viability offered by our model. The relative microbial abundances between time points appeared to change with fermentation. We observed a shift in beta diversity within the first 24 h of fermentation, which stabilized thereafter (Figure 3C). The relative abundances of phyla were modified by using our bioreactor. These included an increase in Actinobacteria and Proteobacteria phyla compared to the beginning of fermentation (Figures 4A, B). Using the Bray–Curtis similarity index, we then compared the replicates of fermentation to assess the reproducibility of our model. When comparing the similarity between each replicate at a given time point, we found that the 24-h replicates were most similar to each other, suggesting that the growth of microbial communities in our bioreactor tends toward the same pattern (Table 1). Hence, our model allowed the growth of most of the human gut microbiota in a reproducible manner.

**Table 1:** Bray–Curtis similarity index for each time point.

	T0	T24	T48
Bray–Curtis similarity index	0.11 ± 0.05^b^	0.44 ± 0.26^a^	0.36 ± 0.07^ab^

Results that do not share the same letter (a,b) are significantly different (*p* < 0.05) from each other.

### 3.3 RS2 progressively modifies gut microbiota composition and SCFA production

We then investigated the capacity of our model to respond to the addition of compounds such as RS2, which are known to modulate the human gut microbiota composition. We followed the same protocol as our previous human microbiota experimentation, but RS2 was added at the beginning of the fermentation. With respect to the number of viable bacteria, we did not observe a change in Ct values between the control and RS2 groups at any time point (Figure 3A). Following RS2 fermentation, Ct values decreased between 0 and 24 h, similar to the control group, revealing that the growth rate was normal and unaffected by treatment in the first 24 h. However, after 48 h, the differences in Ct from the start of fermentation were maintained for the RS2 group. Bacterial alpha diversity, represented by the Shannon index, was not altered by the addition of RS2 (Figure 3B). The addition of our prebiotic shifted gut microbiota composition after 24 h and 48 h of fermentation (Figure 3C). Using LEfSe, we assessed the changes in the gut microbiota between control and RS2 fermentations. At the beginning of fermentation, *Streptococcus thermophilus* was overrepresented in the RS2 fermentation, and *Anaerostipes hadrus*, *Clostridium sensu stricto,* and *Collinsella aerofaciens* were overrepresented in the control fermentation (Figure 4C). After 24 h, we observed an increase in *Bifidobacterium adolescentis, Bifidobacterium faecale, Bifidobacterium* spp., *Bacteroides uniformis, Streptococcus* spp., and *Peptostreptococcus anaerobius* in the RS2 fermentation. For the control fermentation, *Clostridium perfringens, Bifidobacterium bifidum, Bacteroides dorei,* and *Parabacteroides distasonis* were overrepresented compared to the RS2 fermentation (Figure 4D). After 48 h, an increase in *Bifidobacterium adolescentis*, *Bifidobacterium faecale, B. longum,* and *Turicibacter sanguinis* were found in the RS2 fermentation. After 48 h, *Bifidobacterium bifidum, Bacteroides xylanisolvens, Bacteroides ovatus*, *Klebsiella* spp., *Alistipes finegoldii,* and *Parabacteroides merdae* were overrepresented in the control fermentation (Figure 4E).

**Figure 5:**
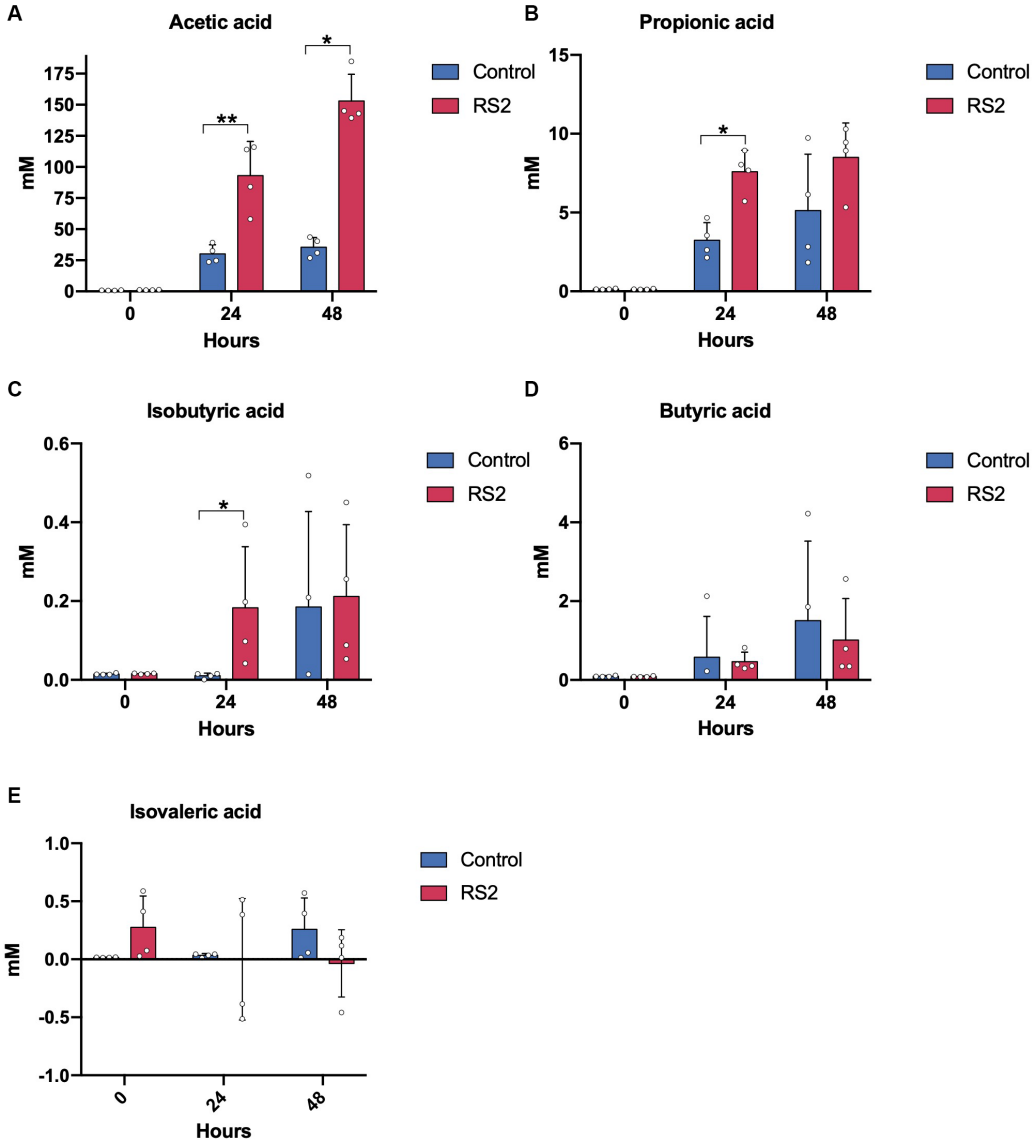
RS2 modulates microbial metabolite production. Levels of SCFAs **(A)** acetic, **(B)** propionic, **(C)** isobutyric, **(D)** butyric, and **(E)** isovaleric acids. **(A–E)** data are represented as means ± s.d. (*n* = 4). Statistical analyses were performed using a repeated two-way ANOVA followed by a Tukey post-hoc test. * = *p* < 0.05, ** = *p* < 0.01.

As SCFAs are primarily derived from the bacterial fermentation of fibers, we next evaluated the impact of RS2 on SCFA levels. Prior to fermentation, no differences in SCFA concentrations were detected between the groups. The supplementation of RS2 significantly increased acetic, propionic, and isobutyric acid levels after 24 h compared with the control fermentation (Figures 5A–C). However, after 48 h of fermentation, only the increase in acetic acid levels remained significant. Butyric and isovaleric acid levels remained similar between the two conditions (Figures 5D–E). These data show that our model has the capacity to reproduce the impact of RS2 in addition to the composition of fecal microbiota and the production of microbial-derived metabolites.

## 4 Discussion

The study aimed to assess the ability of our bioreactor model to provide a favorable environment for the growth of human gut microbiota with a similar functionality. Our findings suggest that our model is capable of offering a reproducible anaerobic environment for the growth of human intestinal microbiota.

Most of the bacterial community was able to grow inside the bioreactor for 48 h with high similarity between replicates. The alpha diversity of the microbiota was not affected by the use of our bioreactor, as no difference was found between the alpha diversities of the inoculum and the fermentation values. Indeed, the alpha diversity values observed in our system are similar to those found in other models (Auchtung et al., 2015; Rui et al., 2019). In terms of beta diversity, we observed a shift with fermentation compared to the beginning of the experimentation, with an increase in the relative abundance in the Proteobacteria phylum and a decrease in the relative abundance in the Firmicutes phylum. Other models observed a similar shift (Auchtung et al., 2015; O’Donnell et al., 2018).

We also showed the functional similarities of our model to the human colon environment by adding potato RS2 to the culture environment. Indeed, many clinical trials have investigated the response of the gut microbiota to resistant starch from potatoes. Similar to our finding, previous studies have reported an increase in primary degraders of starch from the Actinobacteria phylum, such as *Bifidobacterium adolescentis/faecale* (Venkataraman et al., 2016; Baxter et al., 2019; Flowers et al., 2019). *In vitro* experiments were consistent with these results, as it was demonstrated that *B. adolescentis* had the ability to adhere to and degrade potato starch granules (Crittenden et al., 2001; Belenguer et al., 2006). *Ruminococcus bromii,* often cited as a degrader of resistant starch, appears to increase when supplemented with RS2 (Abell et al., 2008; Ze et al., 2012). However, the RS2 source influenced which taxa were amplified. RS2 from maize increases the *R. bromii* population, whereas the response of *R. bromii* to RS2 from potato is not robust and may be individual-dependent (Martínez et al., 2010; Baxter et al., 2019)_._ To the best of our knowledge, we were the first to find an *in vitro* increase in *Turicibacter sanguinis* and *Streptococcus* genera with RS2. In the literature, *Turicibacter* species increased in pigs when given raw potato starch (Sun et al., 2016), and in mice (Herrmann et al., 2018), studies suggest that *Turicibacter* could be a potential starch degrader. As RS2 was not pre-digested with alpha-amylase, we hypothesize that the increase in the *Streptococcus* genus was due to the presence of available amylose, as these bacteria have high amylolytic activities (Bui et al., 2020).

At the beginning of fermentation, we were able to find the genus and species differences between the control and RS2 replicates. Resistant starch cannot be autoclaved, so it may contain bacteria that appear in the LEfSe analysis. In line with this reasoning, *Streptococcus thermophilus* was found in all RS2 samples but only in two control samples.

After 48 h, we observed differences between the growth patterns of RS2 fermentation bacteria compared to control fermentation without decreased bacteria viability. After 48 h, the concentration of nutrients in culture media have been consumed, except for the starch which was added in excess, allowing the growth of starch-degrading bacteria and thus maintaining the number of viable bacteria compared to the start of fermentation. We also found slight differences in bacteria overrepresented after 24 h and 48 h for the fermentation of RS2. Indeed, *Turicibacter sanguinis* was only found to be significantly overrepresented after 48 h, and *Bacteroides uniformis* and *Peptostreptococcus anaerobius* were no longer significantly overrepresented after 48 h. Our SCFA results support the hypothesis that primary starch-degrading bacteria produce lactate, acetate, and monosaccharides from RS2 that can be then utilized by cross-feeder bacteria, thereby promoting their growth. At 24 hours, we observed an increase in acetate originating from primary starch degraders, as well as an increase in propionate and isobutyrate. These latter compounds are probably generated by cross-feeder bacteria utilizing lactate or mono-oligosaccharides. However, after 48 h, other sources of nutrients necessary for the growth of cross-feeders were depleted, and their growth declined resulting in the stabilization of propionate and isobutyrate production. Acetate remained increased, as RS2 was still a source available for primary starch degradation. Consistent with this hypothesis, *Bacteroides uniformis* is known to be capable of producing propionate from succinate (Louis and Flint, 2017), and after 24 h of fermentation, RS2-supplemented bioreactors harbored significantly more *Bacteroides uniformis* than control fermentations. However, after 48 h of fermentation, propionate levels were no longer different from control fermentations, concordant with the absence of overrepresentation of *Bacteroides uniformis* at this time.

Butyrate did not significantly increase with RS2 supplementation, in contrast to previous studies that found an increase in butyrate, following the RS2 consumption in fecal samples (Venkataraman et al., 2016; Alfa et al., 2018; Baxter et al., 2019). However, some studies reported heterogeneity in the production of butyrate due to individual responses (McOrist et al., 2011; Ordiz et al., 2015), with one study showing that microbiota abundant with *B. adolescentis* were less likely to experience an increased butyrate response with potato starch (Baxter et al., 2019). Taken together, the butyrate and *R. bromii* responses suggest that our model could show individual responses to RS2 depending on the inoculum’s donor.

A limitation of our study is that we used the fecal material microbiota from a single donor instead of combining multiple donors. However, it did allow us to identify the potential individual response to RS2. In the future, the response of other donors to RS2 should be investigated.

In summary, we have demonstrated that our low-cost and user-friendly prototype bioreactor model provides a favorable environment for the growth of human gut microbiota and can mimic its response to a prebiotic. It offers the possibility of studying the impact of a specific nutrient on the intestinal microbiota. Our model is modular and can support a variety of applications: the pH target can be changed to simulate another part of the gastrointestinal tract, and semi-continuous functionality (portions of culture media are replaced with fresh culture media at regular intervals) can be added to the model, allowing longer experiments for molecules that take more time to modulate the microbiota. We have also shown that the bioreactor can highlight the individual response to a prebiotic, which opens up new possibilities for applications such as precision nutrition.

## Data availability statement

The datasets presented in this study can be found in online repositories. The names of the repository/repositories and accession number(s) can be found at: European Nucleotide Archive, PRJEB73279.

## Ethics statement

This study uses human fecal microbiota obtained from a biobank sample at the Institute for Nutrition and Functional Foods (INAF) at Laval University. The biobank is composed of materials from human volunteers that is under the management framework for personal information, research material or human biological material (Approval # 2019-219 CG) which is provided by Laval University Health Sciences (LUHS) Committee. This study does not require to be reviewed and approved by the Laval University Human Ethics committee since the study is limited to using biobanked fecal materials from donors that is under the authorization provided by the aforementioned management framework.

## Author contributions

AL-A: Writing – original draft, Conceptualization, Investigation, Formal Analysis. GP: Writing – review and editing, Conceptualization, Visualization. AM: Writing – review and editing, Visualization.
