# Supplementary material for: A low-cost, easy-to-use prototype bioreactor model for the investigation of human gut microbiota: validation using a prebiotic treatment
Source: Front Microbiol. 2024 May 8;15:1250366. doi: 10.3389/fmicb.2024.1250366 (PMC11110930; doi:10.3389/fmicb.2024.1250366)
Supplement: Supplementary file 1 [file Table_1.DOCX]

**Supplementary Table 1: qPCR primer sequences and annealing temperatures**

| **Primer name** | **Target** | **Sequence (5′ - 3′)** | **Reference** |
| --- | --- | --- | --- |
| *BiLON-1* | *Bifidobacterium longum* subsp. *longum* | TTCCAGTTGATCGCATGGTC | (Oki et al., 2018) |
| *BiLON-2* |  | GGGAAGCCGTATCTCTACG | (Oki et al., 2018) |
| *331F* | 16S rRNA | TCCTACGGGAGGCAGCAGT | (Brands et al., 2010) |
| *797R* |  | GGACTACCAGGGTATCTAATCCTGTT | (Brands et al., 2010) |

**References**

Brands, B., Vianna, M. E., Seyfarth, I., Conrads, G., & Horz, H. P. (2010). Complementary retrieval of 16S rRNA gene sequences using broad-range primers with inosine at the 3′-terminus: Implications for the study of microbial diversity. *FEMS Microbiology Ecology*, *71*(1), 157–167. https://doi.org/10.1111/j.1574-6941.2009.00786.x

Oki, K., Akiyama, T., Matsuda, K., Gawad, A., Makino, H., Ishikawa, E., Oishi, K., Kushiro, A., & Fujimoto, J. (2018). Long-term colonization exceeding six years from early infancy of Bifidobacterium longum subsp. longum in human gut. *BMC Microbiology*, *18*(1). https://doi.org/10.1186/s12866-018-1358-6
